# The Significance of Keratinized Mucosa in Implant Therapy: Narrative Literature Review and Case Report Presentation

**DOI:** 10.3390/jcm13123501

**Published:** 2024-06-14

**Authors:** Tomasz Jankowski, Agnieszka Jankowska, Natalia Kazimierczak, Wojciech Kazimierczak, Joanna Janiszewska-Olszowska

**Affiliations:** 1 Private Practice Dental Clinic Jankowscy, Ul. Czerwonego Krzyża 24, 68-200 Żary, Poland; agnieszkajankowska2301@gmail.com; 2 Kazimierczak Private Medical Practice, Dworcowa 13/u6a, 85-009 Bydgoszcz, Poland; natnowicka@gmail.com (N.K.); w.kazimierczak@cm.umk.pl (W.K.); 3 Department of Radiology and Diagnostic Imaging, Collegium Medicum, Nicolaus Copernicus University in Toruń, Jagiellońska 13-15, 85-067 Bydgoszcz, Poland; 4 Department of Interdisciplinary Dentistry, Pomeranian Medical University in Szczecin, 70-111 Szczecin, Poland; jjo@pum.edu.pl

**Keywords:** dental implants, periodontitis, peri-implantitis, free gingival graft, case report, oral surgery

## Abstract

**Background/Objectives**: Implant treatment in patients who require teeth extraction due to periodontitis presents a significant challenge. The consideration of peri-implantitis is crucial when planning the placement of dental implants. The predictability of implant treatment relies on the suitability of both hard and soft tissue quality. The aim of this article is to present a case report demonstrating a secure treatment protocol for implant procedures in patients with periodontitis requiring the extraction of all teeth, soft tissue management targeted at increasing the keratinized mucosa zone, and the provision of a reliable prosthetic solution. The secondary objective is to review the relevant literature regarding the significance of keratinized mucosa surrounding dental implants and its association with the occurrence of peri-implantitis. **Case presentation**: A 65-year-old female with generalized periodontitis, stage IV grade C and very poor oral hygiene came for treatment and rehabilitation of the lower jaw. CBCT revealed periodontal lesions and labio-lingual ridge dimensions in the region of teeth 34–44 from 8.0 to 10.2 mm. The first surgery included teeth extraction and periodontal lesions enucleation with simultaneous placement of four implants in the positions of teeth 32, 34, 42, 44. The second-stage surgery involved increasing the keratinized mucosa using two free gingival grafts. **Conclusions**: The present case report described the treatment process of the patient with periodontitis, including immediate implantation in the infected region, soft tissue augmentation using free gingival grafts and the ultimate placement of a bar-retained overdenture for final restoration. After two years of observation, despite questionable hygiene, no symptoms of gingival inflammation were detected. Furthermore, there is limited information in the literature regarding the correlation between inadequate keratinized gingiva and the occurrence of peri-implantitis.

## 1. Introduction

“Periodontal disease” refers to a variety of persistent inflammatory disorders that affect the gums, bone and collagen fibers of the connective tissue responsible for anchoring the tooth (ligament) to the alveolar bone. It begins with the bacteria present in dental plaque, recognized as a microbial biofilm developing on both the teeth and the gingiva. Untreated gingival inflammation can advance to periodontitis, characterized by the loss of gingiva, bone and ligament, resulting in the formation of deep periodontal pockets. These pockets are a characteristic feature of the disease and may ultimately result in tooth loss [1].

Dental implant treatment in current dentistry is the standard method of the functional and esthetic rehabilitation of missing teeth. Implants serve as a versatile tool for clinicians, enabling them to address a spectrum of dental restoration needs, from replacing a solitary missing tooth to rebuilding an entire edentulous arch.

Implant therapy in patients requiring teeth extraction due to periodontitis is always a great task. Patients with a history of periodontitis and non-compliant with supportive periodontal care are at higher risk of biological complications and implant loss [2].

The biggest challenge in current-day implantology is peri-implant diseases, which is a very common condition. Peri-implantitis is described as a pathological state that arises in the tissues surrounding dental implants. It is marked by inflammation within the connective tissue around the implant and progressive bone loss, extending beyond the normal physiological adaptation period [3]. Besides a prior occurrence of periodontal disease, smoking, inadequate oral care and systemic diseases, the absence of keratinized mucosa (KM) surrounding the implant is mentioned as a contributing factor to peri-implantitis [4].

Currently, there are three main techniques of surgical interventions enlarging the zone of KM: (1) an apically positioned flap alone (APF), (2) an APF plus a free gingival graft (FGG), (3) an APF plus a collagen matrix [5]. Thoma et al. [5] concluded that an APF plus autogenous graft resulted in a greater improvement of bleeding indices and higher marginal bone levels than other techniques.

Bassetti et al. [6], considering the limitations of their review, concluded that depending on the localization and the clinical situation, different surgical augmentation techniques of KM can be used. The maxilla apically positioned partial-thickness flap (APPTF) to gain KT and the roll envelope flap (REF) to gain soft tissue volume are efficacious and minimally invasive techniques. In the mandible, to gain KT, the employment of APPTF in combination with a free gingival graft (FGG) or a xenogeneic graft material (XCM) are both highly predictable, although soft tissue substitutes increase the expense of the treatment.

The purposes of the present article are as follows: (1) to present a case report of safe treatment protocol for patients with periodontitis requiring all teeth extraction, soft tissue management using free gingival grafts and predictable prosthetic solution on dental implants for the mandible; (2) to review the relevant literature concerning the significance of keratinized mucosa around dental implants and its association with the development of peri-implantitis.

## 2. Study Selection and Review of the Literature

**Figure 1:**
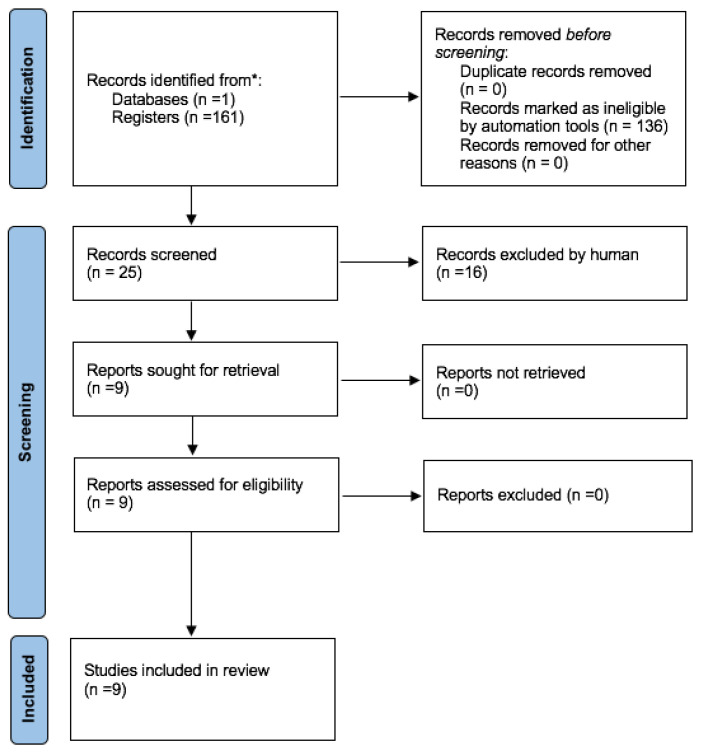
Prisma 2020 flow diagram outlining the process of selecting studies for the literature review. * Consider, if feasible to do so, reporting the number of records identified from each database or register searched (rather than the total number across all databases/registers).

A review of the literature was performed to check the state of knowledge regarding the association between the lack of KM and peri-implantitis. The search was conducted on 11 May 2024 in the most popular database—PubMed (Medline). The following keywords were used during the search process: “peri-implantitis” AND (“keratinized mucosa” OR “attached mucosa” OR “attached gingiva” OR “free gingival graft” OR “apically positioned partial-thickness flap”). To ensure the highest quality of review, only cohort studies, case-control studies and randomized clinical trials were included in the search. Nevertheless, no suitable records were found, and therefore, meta-analyses and systematic reviews were included in this literature review, which is ultimately narrative in nature. The flow diagram is presented in Figure 1 (Prisma 2020 flow diagram).

The studies selected for the review examined the association between the keratinized mucosa (KM) and the occurrence of peri-implantitis. Publications referring to a non-human species were excluded. Finally, nine systematic reviews or systematic reviews and meta-analyses were identified (Table 1). The keywords used in the study selection did not allow for finding any other types of research.

## 3. Case Report

### 3.1. Patient Information, Diagnosis and Treatment Plan

**Figure 2:**
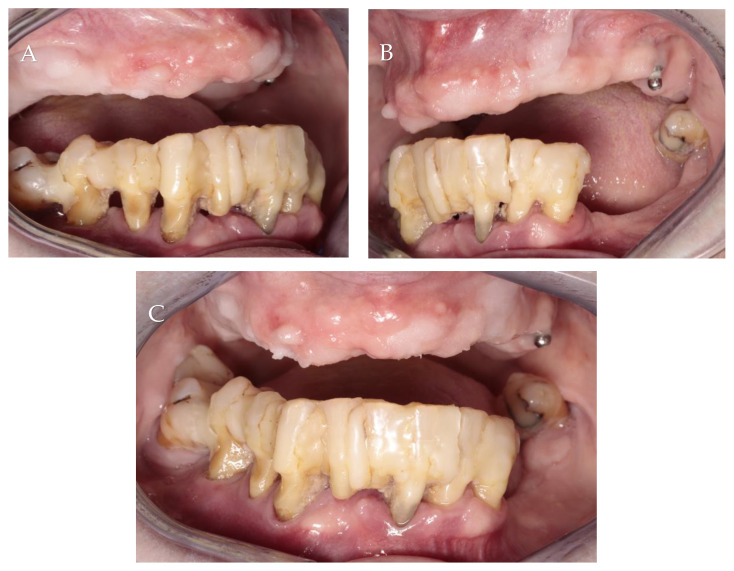
Initial intraoral photographs: (**A**) right lateral view; (**B**) left lateral view; (**C**) frontal view.

A 65-year-old non-smoking female with a medical history of hypertension and hypothyroidism came to Private Practice Dental Clinic Jankowscy in Żary for treatment and rehabilitation of the lower jaw. Intraoral examination showed generalized periodontitis, stage IV grade C, according to a manuscript released by Caton et al. [15]. All teeth were connected by composite, except wisdom teeth. The patient had very poor oral hygiene. She was using an upper complete denture stabilized on one implant in the position of tooth 26. Her chief complaint was pain, gingival bleeding and the inability to eat solid food (Figure 2).

**Figure 3:**
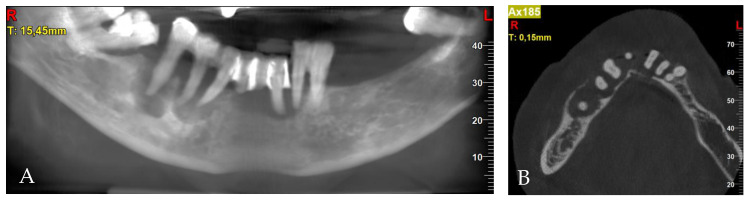

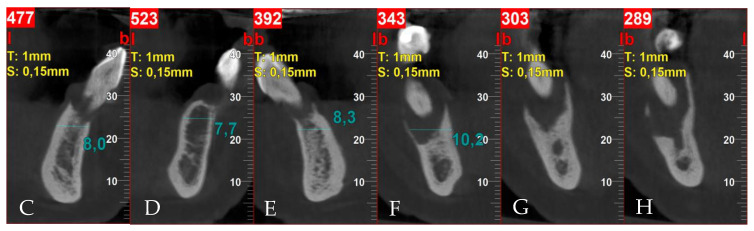
Pre-treatment CBCT: (**A**) panoramic view; (**B**) axial view; (**C**) cross-sections in position of teeth, respectively, 32; (**D**) 34; (**E**) 42; (**F**) 44; (**G**) 45; (**H**) 45.

After clinical examination, the 3D cone-beam computed tomography (CBCT) was taken. Periodontal lesions from teeth 31, 32, 43, 44 and 45 were diagnosed. The biggest one from tooth 45 was very close to the mental nerve. CBCT confirmed the necessity of all-teeth extraction. Labio-lingual ridge dimensions in the region of teeth 34–44 were from 8.0 to 10.2 mm (Figure 3).

The decision to proceed with immediate implant placement was determined by the quality and quantity of the remaining bone, which supported this approach. Lee et al. [16], in a systematic review and meta-analysis, concluded that placing implants immediately in extraction sockets with periapical infections is viable with careful debridement and adherence to appropriate surgical protocols. Several investigations uphold this point of view [17,18,19]. Consequently, after oral hygiene education, the treatment plan was settled as follows:

- Extraction of hopeless teeth and enucleation of periodontal lesions with immediate implantation in the positions of teeth 32, 34, 42, 44 and bone augmentation;
- After two months of evaluation of soft tissues and possible augmentation of them using free gingival graft/grafts;
- Bar retained overdenture as the final restoration.

### 3.2. Surgery 1—Teeth Extraction and Periodontal Lesion Enucleation with Simultaneous Implantation

**Figure 4:**
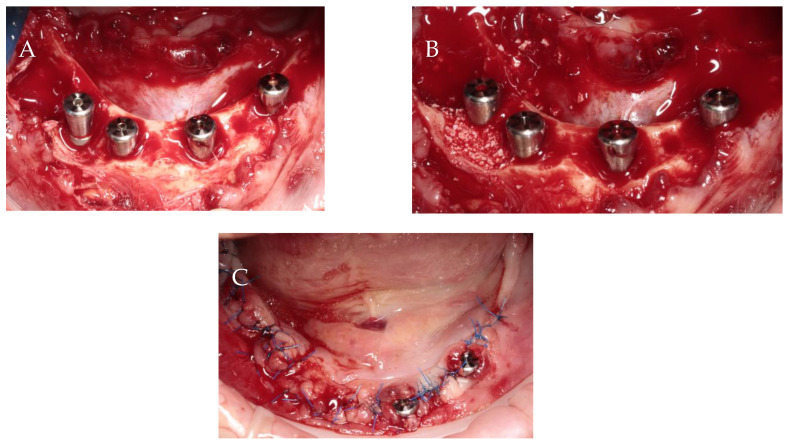
Surgical procedure: (**A**) implant insertion; (**B**) bone augmentation; (**C**) flap repositioning and suturing.

**Figure 5:**
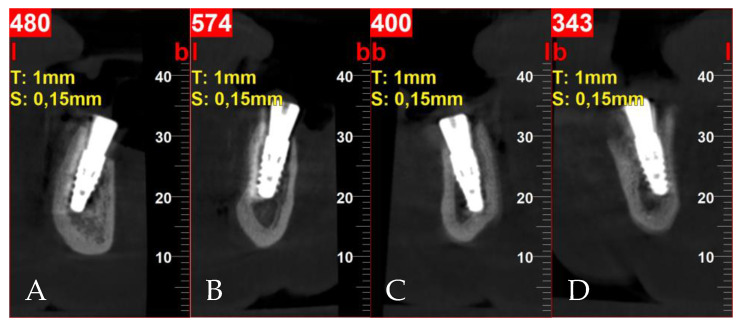
Postoperative cross-sectional CBCT scans: (**A**) tooth 32; (**B**) tooth 34; (**C**) tooth 42; (**D**) tooth 44.

Implant size and location were established using iRYS software (version 10.0) for CBCT radiographs (MyRay, Hyperion X5 3D, Cefla s.c., Imola, Italy). Preoperative antibiotic prophylaxis includes administering Clindamycin 600 mg one hour prior to surgery, followed by a postoperative regimen of 300 mg every eight hours for six days. For economic reasons, the temporary restoration patient chose a removable full denture. The surgery was executed under local anesthesia using articaine with adrenaline (Dentocaine 40 mg/mL + 0.01 mg/mL; Laboratorios Inibsa, S.A.; Lliçà de Vall, Spain). All teeth were extracted, and full-thickness buccal and lingual flaps were performed. In order to achieve better conditions for implantation, alveolar osteoplasty was performed. With great precaution due to the proximity of the mental nerve, the periodontal lesions were enucleated. Four implants with a diameter of 3.75 mm and a length of 10 mm were inserted 1 mm subcrestal in relation to the buccal bone (ICX-Active Master implant, Medentis medical, Bad Neuenahr-Ahrweiler/Germany). Primary stability was 34 Ncm, 10 Ncm, 30 Ncm and 28 Ncm for implants in the position of teeth 32, 34, 42 and 44, respectively. Moreover, 6 mm high healing screws were fixed to the implants. Bone defects resulting from periodontal lesions removal were augmented by bovine-derived xenograft (Bio-Oss 0.25–1 mm; Geistlich; Wolhusen, Switzerland) mixed with autogenous bone collected from the left side of the mandible using a scraper, outside implantation area. The flaps were repositioned using simple non-resorbable 5/0 nylon sutures (Seralon, Serag-Wiessner, Naila, Germany) (Figure 4). After surgery, CBCT evaluation was assessed (Figure 5).

In addition to antibiotic prevention, the patient was advised to take non-steroidal anti-inflammatory drugs and rinse with a 0.1% chlorhexidine digluconate mouthwash. Two weeks after surgery, healing was progressing properly and all sutures were removed.

**Figure 6:**
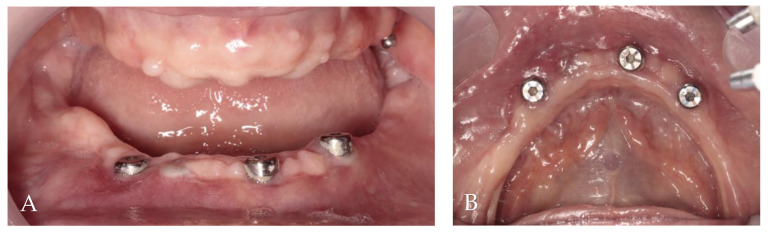
Two months post-op intraoral photographs: (**A**) frontal view; (**B**) occlusal view.

After two months, a clinical assessment revealed a lack of KM on the labial side of all implants. The implant with a healing screw in position for tooth 42 was beneath soft tissues. The decision to perform two free gingival grafts from the palate around all implants was made (Figure 6).

### 3.3. Surgery 2—Apically Positioned Partial-Thickness Flap (APPTF) in Combination with Free Gingival Grafts (FGGs)

Following the procedure, the patient received standard antibiotic coverage with 2 g Amoxicillin 1 h before the surgical procedure, followed by 500 mg taken three times daily for one week.

After the administration of local anesthesia utilizing articaine with adrenaline (Dentocaine 40 mg/mL + 0.01 mg/mL; Inibsa Dental S.L.U.; Spain), 1 mm depth incision along the mucogingival junction on the buccal side of the implants was made. Two vertical incisions, each measuring 10 mm, were made through the alveolar mucosa at the ends of the horizontal incision. A flap of partial thickness was elevated and positioned apically using simple resorbable 5/0 polyglycolic acid sutures attached to the periosteum (PGA, Atramat, Ciudad de México, Mexico). From both sides of the hard palate in the region from the canine to the first molar of the maxilla, two free gingival grafts of a thickness of 1.5 mm and dimensions of 6/25 mm were collected according to the measurements made by the periodontal probe.

**Figure 7:**
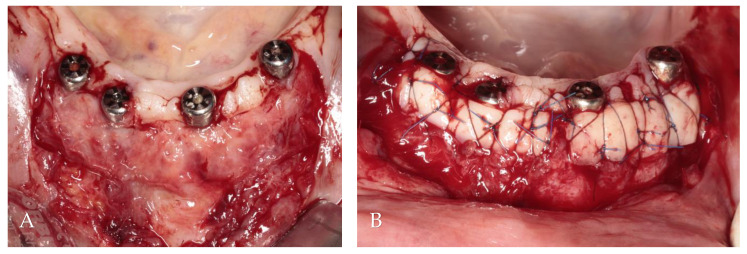
Soft tissue augmentation procedure: (**A**) preparation of the recipient site; (**B**) free gingival grafts stabilized by mattress sutures between residual KM and periosteum.

Subsequently, the grafts were placed on the recipient side and initially stabilized by simple 5/0 nylon sutures (Seralon, Serag-Wiessner, Germany) between grafts and residual attached gingiva. Non-resorbable 6/0 nylon mattress sutures (Seralon, Serag-Wiessner, Germany) were employed to press the grafts to the periosteum (Figure 7).

**Figure 8:**
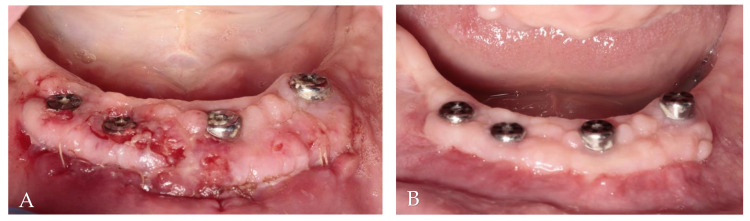
Clinical evaluation of soft tissue healing: (**A**) 2 weeks after augmentation; (**B**) 2 months after augmentation.

The donor sites were protected by a gelatin sponge stabilized by 5/0 nylon sutures (Seralon, Serag-Wiessner, Germany). As after the first surgery, non-steroidal anti-inflammatory drugs and 0.1% chlorhexidine digluconate mouthwash were recommended. The sutures were removed after 14 days. Then, 2 months after free gingival grafts, prosthetic treatment began, and it lasted 3 weeks (Figure 8).

### 3.4. Results

**Figure 9:**
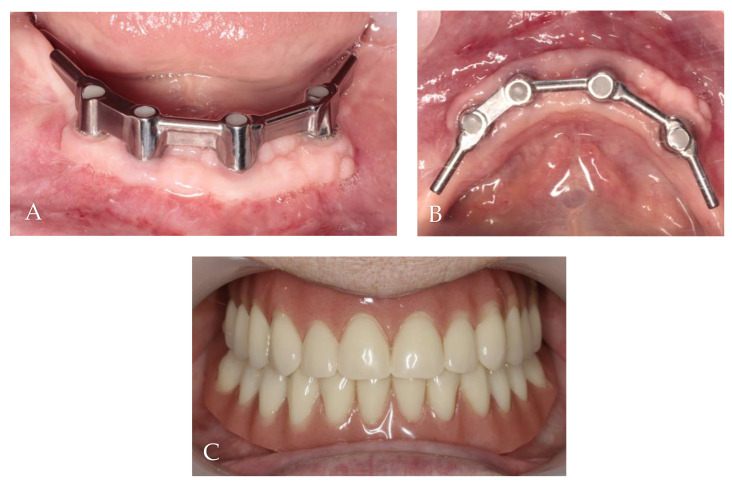

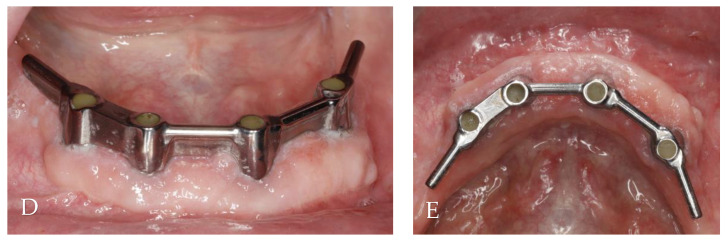
Follow-up clinical photographs: (**A**–**C**) 4 months after first surgery—the prosthetic restoration’s finalization; (**D**,**E**) 24 months after bar-retained overdenture delivery.

**Figure 10:**
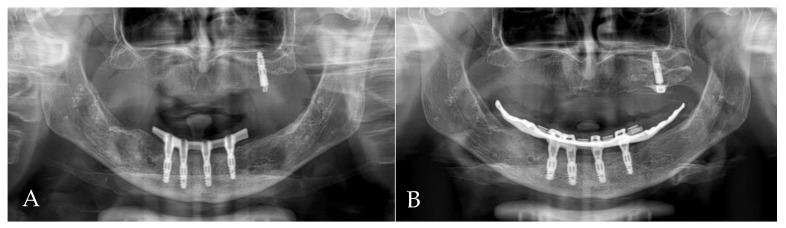
Orthopantomogram (OPG): (**A**) 4 months after first surgery—the prosthetic restoration’s finalization; (**B**) 24 months after bar-retained overdenture delivery.

The first follow-up was conducted after 1 month, and the last one occurred after 24 months. All implants survived, and KM thickness increased on the buccal side by approximately 2 mm (Figure 9). The functionality of the mental nerve remained intact. Under the bar, plaque was detected; nonetheless, no symptoms of peri-implant soft tissue inflammation were observed. All implants presented a probing depth from 2 to 3 mm, and there was no bleeding on probing. Moreover, the orthopantomogram revealed no bone resorption (Figure 10). Patient oral hygiene education was repeated.

## 4. Discussion

The association between KM and peri-implant tissue health has been the subject of research for many years. However, there are no studies in the literature other than systematic reviews and meta-analyses regarding a direct correlation between the width of keratinized mucosa and the occurrence of peri-implantitis.

This narrative review revealed that three of nine studies [7,9,13] identified the lack of keratinized mucosa as a risk factor for developing peri-implantitis. Brito et al. [10] concluded that the presence of an appropriate zone of keratinized tissue results in better peri-implant tissue health, which is consistent with the research of Thoma et al. [5] and Stefanini et al. [14]. Carra et al. [11] recognized KM augmentation procedures as favorable for the control of peri-implant inflammation and the stability of marginal bone levels. In contrast to previous studies, Dreyer et al. [12] stated that there is no convincing evidence available that the absence of keratinized mucosa (KM) is one of the risk factors for peri-implantitis. According to this systematic review, smoking, diabetes mellitus, inadequate prophylactic measures, and a history or existing condition of periodontitis are the primary risk factors most crucial for peri-implantitis. In addition, Revida et al. [8] concluded that the influence of keratinized mucosa width on the development of peri-implantitis appears to be negligible.

Subsequently, using only the keyword “keratinized mucosa” in PubMed (Medline), it is possible to find many other studies describing the influence of KM presence on soft tissue health surrounding the implant. Most investigators agree that KM is crucial for peri-implant health. The authors consider the importance of the KM in terms of the following parameters: gingival index score, plaque index score (plaque accumulation), bleeding scores, radiographic bone loss, periodontal attachment loss and mucosal recession. In a cross-sectional study, Bouri et al. [20] declared that implants with a lack of keratinized mucosa (<2 mm) had a significantly higher mean gingival index score, plaque index score and radiographic bone loss. Schrott et al. [21] examined 386 mandibular dental implants placed in 73 completely edentulous patients and restored with fixed full-arch prostheses. They concluded that the lack of adequate KM (<2 mm) had an influence on plaque accumulation and bleeding scores; however, this was only on lingual sites. In Adibrad et al.’s study [22] on 66 functioning dental implants supporting overdentures, the mean gingival index score, plaque index score and bleeding on probing were significantly higher for those implants with a narrow zone (<2 mm) of KM. Moreover, implants with a wider KM zone (≥2 mm) were related to less periodontal attachment loss and mucosal recession [22]. Zigdon et al. [23] also explored the impact of keratinized mucosa (KM) on the condition of the tissues surrounding the implant. They assumed that a wider mucosal band (>1 mm) was associated with less mucosal recession compared with a narrow (<1 mm) band. Also, a wider KM band was associated with greater probing. Consequently, the authors concluded that the KM around dental implants affects both the clinical and the immunological parameters at these sites.

Lin et al. [24], in their systematic review and meta-analyses, extracted mainly from cross-sectional studies, stated that the presence of at least 1–2 mm wide KM might be favorable in decreasing plaque accumulation, tissue inflammation, attachment loss, and mucosal recession. Chung et al. [25] stated that the absence of adequate keratinized mucosa (KM) or attached mucosa (AM) in dental implants, especially in posterior implants, is associated with higher plaque accumulation and gingival inflammation but not with more annual bone loss, regardless of their surface configurations.

Oh et al. [26] examined free gingival grafts and their impact on crestal bone loss around implants. They stated that FGG for implants exhibiting a lack of KM is a viable treatment option to reduce mucosal inflammation and to maintain crestal bone level in the short term. Kim et al. [27] reached different conclusions regarding the impact of KM on hygiene around implants. They found that in cases with a lack of KM in the vicinity of implants, its deficiency does not necessarily have an adverse effect on hygiene management and soft tissue conditions. Nevertheless, the risk of an increase in gingival recession and crestal bone loss is present. Therefore, it is thought that from the aspect of long-term preservation and management, as well as for the area demanding esthetics, the presence of an appropriate volume of keratinized gingiva is required [27]. Furthermore, Fons-Badal et al. [28] noticed that after non-surgical treatment, implants surrounded by keratinized gingiva demonstrated superior outcomes.

A history of periodontal disease is also mentioned as one of the risk factors for peri-implantitis. Derks et al. [29] clinically and radiographically examined 588 patients who all had received implant-supported therapy 9 years earlier. They reported that 45.0% of all patients presented with peri-implantitis (bleeding on probing and bone loss >0.5 mm), and 14.5% of diagnoses were severe peri-implantitis (bleeding on probing and bone loss >2 mm). Furthermore, they concluded that patients with periodontitis and with ≥4 implants, as well as prosthetic therapy delivered by general practitioners, presented higher odds ratios for moderate peri-implantitis. A systematic review by Carra et al. [30] concluded that in partially edentulous patients receiving an implant-supported fixed partial denture, a history of periodontitis is associated with a poorer survival rate and an increased risk of peri-implantitis over a 5–10-year follow-up period after implant loading.

Moreover, narrow KM is sometimes related to muscle pull due to high frenum attachments or a shallow vestibule [31].

## 5. Conclusions

(1) Many authors agree that keratinized mucosa is essential for maintaining peri-implant health. Nevertheless, there are different opinions about the specific impact of KM on various parameters describing the condition of the tissues in the vicinity of implants.
(2) There is insufficient knowledge in the literature regarding the correlation between inadequate KM and the occurrence of peri-implantitis. Further research in this area is needed, especially case–control studies, cohort studies and randomized clinical trials.
(3) A history of periodontitis is mentioned as one of the risk factors for peri-implantitis.
(4) The present case report described the treatment process of a patient with periodontitis, including immediate implantation in the infected region, soft tissue augmentation using free gingival grafts and the ultimate placement of a bar-retained overdenture as the final restoration. After two years of observation, despite questionable hygiene, no symptoms of gingival inflammation were detected. The orthopantomogram did not show any bone resorption.

## Figures and Tables

**Table 1:** Characteristics of selected studies.

Author, Year of Publication	Number of Included Studies	Results	Conclusions
1. Thoma et al., 2018 [5]	10	Surgical interventions aimed at augmenting keratinized tissue demonstrated a notably superior enhancement in gingival index values when compared to both groups with or without keratinized tissue.	Soft tissue grafting techniques lead to improved peri-implant health outcomes.
2. Mahardawi et al., 2023 [7]	22 (16 were meta-analyzed)	The overall analysis revealed that the absence of keratinized mucosa correlated with an increased occurrence of peri-implantitis.	Insufficient keratinized mucosa is a risk factor, elevating the prevalence of peri-implantitis and highlighting the necessity of consideration during dental implant placement.
3. Ravidà et al., 2022 [8]	9 (4 in the meta-analysis)	There were no notable differences between groups, and limited evidence was found for probing depth, marginal bone loss and soft-tissue recession. However, a significant advantage was observed for a keratinized mucosa width (KMW) of at least 2 mm, with a lower mean plaque index, as indicated by three studies involving (430 implants, low-quality evidence).	The impact of keratinized mucosa width (KMW) as an underlying factor for peri-implantitis remains minimal.
4. Ramanauskaite et al., 2022 [9]	22 articles (21 studies)	Peri-implant mucositis and peri-implantitis were observed in 20.8% to 42% and at 10.5% to 44% of the implants with less than 2 mm or absent (0 mm). Conversely, at implant sites with a KT width of at least 2 mm or more than 0 mm, the corresponding values were 20.5% to 53% and 5.1% to 8%, respectively.	Inadequate KT width is correlated with heightened occurrences of peri-implantitis, plaque accumulation, soft-tissue inflammation, mucosal recession, marginal bone loss and greater patient discomfort.
5. Brito et al., 2014 [10]	7	Bleeding indexes were noted in five studies overall, with three studies showing a clear association between higher bleeding indexes of implants with a perimeter of less than 2 mm. Additionally, five studies found significantly higher plaque scores in areas with insufficient KM width (<2 mm), while one study found no notable differences between sufficient and insufficient KM widths.	Having enough keratinized tissue around implants might be crucial as it has been demonstrated to correlate with better peri-implant tissue health.
6. Carra et al., 2023 [11]	48	Dental implants inserted in sites with augmented peri-implant keratinized mucosa (PIKM) tend to exhibit reduced inflammation and lower marginal bone level (MBL) changes (compared to implants with inadequate PIKM).	Augmentation procedures for peri-implant keratinized mucosa (PIKM) in cases of deficiency may promote better management of peri-implant inflammation and help maintain stability in marginal bone levels.
7. Dreyer et al., 2018 [12]	57	There is not sufficient convincing or strong evidence available to suggest that the absence of keratinized mucosa is a significant risk factor for peri-implantitis.	The overall level of evidence to answer the research question is considered weak. Future studies, ideally prospective, randomized and controlled with adequate sample sizes, are necessary to provide more information.
8. Afrashtehfar et al., 2023 [13]	22 (16 were meta-analyzed)	Research specifically targeting fixed prostheses showed correlation between the absence of keratinized mucosa and a higher incidence of peri-implantitis. This association remains significant even in patients receiving regular implant maintenance. Studies that accounted for other variables also reinforced the elevated risk of peri-implantitis in cases of insufficient keratinized mucosa.	The lack of KM was found to elevate the risk of peri-implantitis, underscoring the importance of taking it into account when placing dental implants.
9. Stefanini et al., 2023 [14]	15	The majority of the enhanced implant sites sustained peri-implant health over the medium and long term, with the occurrence of peri-implant mucositis ranging from 0% to 50% and peri-implantitis from 0% to 7.14%.	Implants that received soft tissue augmentation procedures demonstrated a consistently high overall survival rate and a relatively low occurrence of peri-implantitis in the medium and long term.

## Data Availability

No new data were created or analyzed in this study. Data sharing is not applicable to this article.
